# Influence of environmental factors on the abundance of *Anopheles farauti *larvae in large brackish water streams in Northern Guadalcanal, Solomon Islands

**DOI:** 10.1186/1475-2875-10-262

**Published:** 2011-09-13

**Authors:** Hugo Bugoro, Jeffery Hii, Tanya L Russell, Robert D Cooper, Benny KK Chan, Charles Iro'ofa, Charles Butafa, Allen Apairamo, Albino Bobogare, Cheng-Chen Chen

**Affiliations:** 1 Institute of Tropical Medicine, National Yang-Ming University, No. 155, Sec.2, Li-Nong Street, Taipei 112, Taiwan; 2 National Vector Borne Disease Control Programme, Ministry of Health, Honiara, Solomon Islands; 3 Malaria, Other Vector-Borne and Parasitic Diseases, Regional Office for the Western Pacific, World Health Organization, San Lazaro Hospital Compound, Manila, Philippines; 4 Pacific malaria Initiative Support Center, Australian Center for Tropical and International Health, School of Population Health, The University of Queensland, Herston, 4006, Australia; 5 Australian Army Malaria Institute, Gallipoli Barracks, Enoggera, 4052, Australia; 6 Research Centre for Biodiversity, Academia Sinica, 128 Sec 2, Academia Road, Taipei 115, Taiwan

## Abstract

**Background:**

The main vector of malaria in Solomon Islands is *Anopheles farauti*, which has a mainly coastal distribution. In Northern Guadalcanal, Solomon Islands, high densities of *An. farauti *are supported by large brackish streams, which in the dry season are dammed by localized sand migration. The factors controlling the high larval productivity of these breeding sites have not been identified. Accordingly the influence of environmental factors on the presence and density of *An. farauti *larvae was assessed in three large naturally dammed streams.

**Methods:**

Larval sites were mapped and anopheline larvae were collected monthly for 12 months (July 2007 to June 2008) from three streams using standard dippers. Larval collections were made from 10 locations spaced at 50 m intervals along the edge of each stream starting from the coast. At each collection point, floating filamentous algae, aquatic emergent plants, sun exposure, and salinity were measured. These environmental parameters along with rainfall were correlated with larval presence and density.

**Results:**

The presence and abundance of *An. farauti *larvae varied between streams and was influenced by the month of collection, and distance from the ocean (*p < *0.001). Larvae were more frequently present and more abundant within 50 m of the ocean during the dry season when the streams were dammed. The presence and density of larvae were positively associated with aquatic emergent plants (presence: p = 0.049; density: p = 0.001). Although filamentous algae did not influence the presence of larvae, this factor did significantly influence the density of larvae (p < 0.001). Rainfall for the month prior to sampling was negatively associated with both larval presence and abundance (p < 0.001), as high rainfall flushed larvae from the streams. Salinity significantly influenced both the presence (p = 0.002) and density (p = 0.014) of larvae, with larvae being most present and abundant in brackish water at < 10‰ seawater.

**Conclusion:**

This study has demonstrated that the presence and abundance *An. farauti *larvae are influenced by environmental factors within the large streams. Understanding these parameters will allow for targeted cost effective implementation of source reduction and larviciding to support the frontline malaria control measures i.e. indoor residual spraying (IRS) and distribution of long-lasting insecticidal nets (LLINs).

## Background

In the Solomon Islands, malaria is transmitted by members of the *Anopheles punctulatus *group, including *Anopheles farauti, Anopheles punctulatus *and *Anopheles koliensis *[1]. Of these, *An. punctulatus *and *An. koliensis *have become uncommon and with limited distributions due to the past malaria eradication and control campaigns using indoor residual spraying (IRS) and the distribution of insecticide-treated nets (ITNs) [2-4]. However, *An. farauti *changed its feeding behaviour from late night to outdoor early evening allowing it to avoid the insecticide [5]. *An. farauti *has become the major vector [1,6,7] and the early outdoor feeding behavior of *An. farauti *reduces the efficacy of IRS and ITNs; therefore, additional complementary vector control tools which target other stages of the mosquito life-cycle are needed, one example being larviciding.

*An. farauti *breeds both in fresh water and brackish with water up to 70% seawater [8-10]. *An. farauti *has been found to breed in a variety of fresh water filled depressions either natural or man-made such as drains, vehicle tracks, foot prints, pig wallows and borrow pits [11]. These sites are small and suffer the vagaries of rainfall, continually drying out or being flushed out; the adult output from these types of sites is low [8,12]. In the coastal areas of the Solomon Islands very high densities of *An. farauti *are maintained due to the presence of large, permanent, brackish water streams and swamps that form, during the dry season, behind sand bars which block the flow of water into the sea [8,12-14]. As many as 32 permanent coastal streams and swamps have been identified in the northern part of Guadalcanal [15,16]. Previous studies have recorded high adult biting densities and parasite rates in the villages co-located with these coastal streams and swamps [4,17].

Recent empirical [18,19] and theoretical [20] studies have demonstrated that in situations where the proportion of indoor exposure to mosquito bites is less than 50%, the level of protection provided by IRS and LLINs, although still valuable, is significantly reduced. In such situations, additional control measures, i.e. larval control, should be considered when developing integrated vector control programmes to complement the use of IRS and LLINs. To support potential larviciding operations, knowledge of the environmental, biotic and climatic factors that regulate the presence and abundance of *An. farauti *in these breeding sites will enable programme managers to make evidence-based decisions that will best achieve the desired malaria control outcomes. The present study was carried out to determine the association of environmental factors with larval presence and abundance from three large coastal brackish water streams in North Guadalcanal and to discuss the implications for malaria control.

## Methods

### Study site

**Figure 1:**
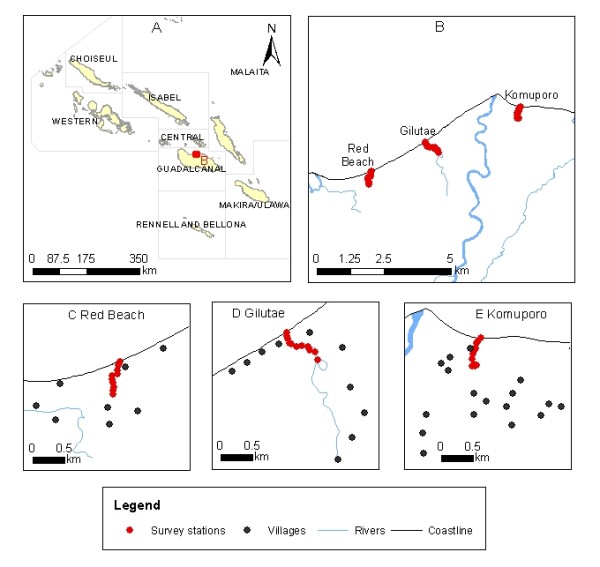
**Map of the study sites.** (A) Map of the Solomon Islands indicating the study area on Guadalcanal province. (B) North Guadalcanal showing the three study sites. (C, D, E) The relative locations of each of the dammed brackish water stream and surrounding villages.

**Figure 2:**
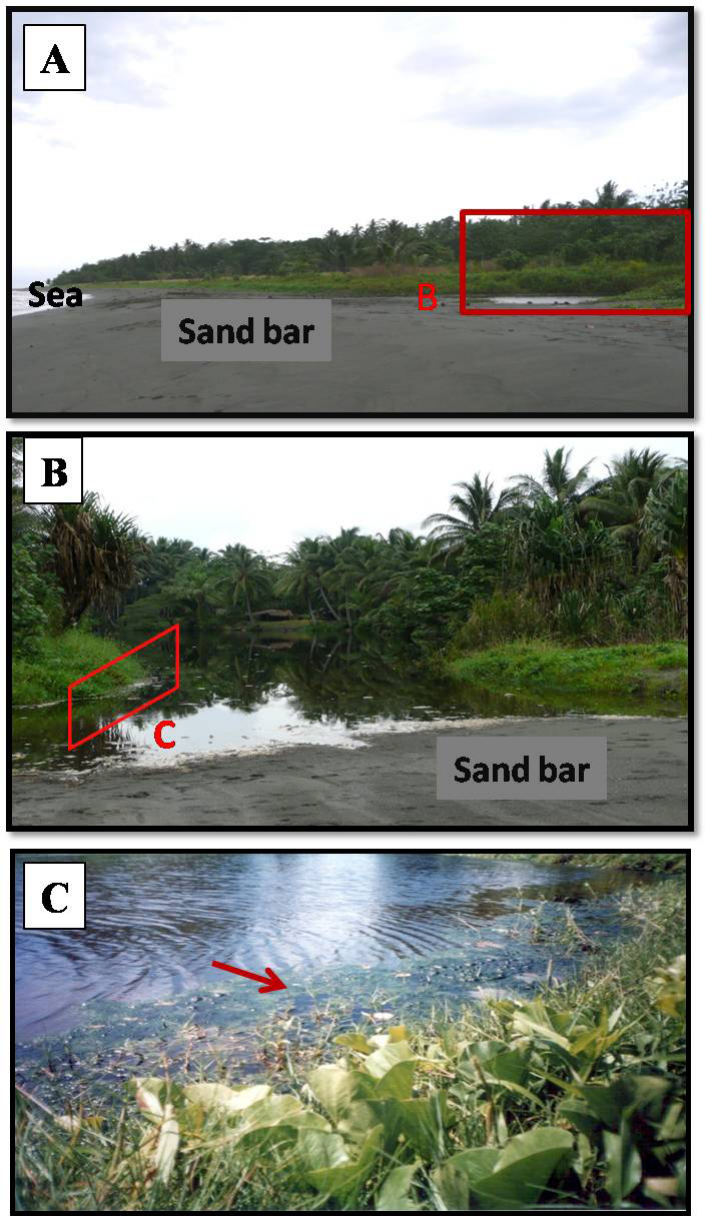
**Typical dammed brackish water stream.** (A) Lateral view of the dammed stream separated from the sea by sand bar. (B) Showing dammed stream. (C) Floating filamentous algae on dammed brackish water stream margin as indicated by arrow.

The study was conducted at three coastal streams at: Red Beach (9.255883° S, 160.062177° E), Gilutae (9.421717° S, 160.131569° E) and Komuporo (9.411026° S, 160.160111° E) (Figure 1) on the north coast of Guadalcanal Province from July 2007 to June 2008 (no collections were made in February of 2008 due to heavy rain and flooding). These large streams are approximately 5 km apart and measure 10-20 m in width with a length ranging from 500 m at Red Beach to up to 1,000 m at Gilutae and Komuporo. The criteria used in selecting the three sites for this study were a) a periodic sandbar blocking the outflow of water to the sea, producing a pool of stagnant water (Figure 2 A, B) and 2b) high levels of *An. farauti *breeding in the large streams. The communities adjacent to these brackish water streams are rural settlements scattered throughout the surrounding bushland on an extensive coastal plain (Figure 1), who experience intense year-round malaria transmission [4].

### Larval collections

Monthly larval collections were made along the margin of the streams from the sea end to about 450 m inland. For each site, 10 sampling stations were set at 50 m apart, giving a total of 30 edge habitat sites (Figure 1). Each station was sampled using standard larval dippers (350 ml), 10 dips per site were taken from different locations with a 1 × 5 m sampling station along the edge of each stream. The collected larvae were counted, and 3^rd ^and 4^th ^instar larvae were reared through to adults which were identified by morphology using standard taxonomic keys [1]. A sample of these mosquitoes were desiccated on silica gel and transported to the National Yang-Ming University in Taiwan for molecular verification. The isomorphic species composition of the *An. farauti *complex was verified by polymerase chain reaction - restriction fragment length polymorphism (PCR-RFLP) using the internal transcribed spacer region 2 of the ribosomal DNA. The analysis, DNA extraction, amplification, restriction digest, fragment separation, and visualization were as previously described [21].

### Estimation of environmental factors

The percentage cover of filamentous algae and aquatic emergent plants was estimated using the method described by Mckenzie *et al *[22]. The percentage cover of filamentous algae, aquatic emergent plants was estimated using a 1 × 1 m quadrat within each 1 × 5 m sampling station. The quadrat, which was made from 4 × 1 m steel wires and floatable polystyrene foam, was placed systematically at four different places and spaced 25 cm apart along the length of the sampling station. Using a digital camera (Pentax, model K100D) photographs were taken from an angle as vertical as possible on the entire quadrat frame and corresponding quadrat label, taking care to avoid any shadows or patches of reflection off any water in the field of view. The total percentage cover of emergent plant vegetation was estimated by drawing the outline of any areas of algal cover onto tracing paper superimposed on the photographs and averaging the shaded area as a proportion of the total surface area using quadrat grids.

The percentage of sunlight was estimated using the method described by Nichols *et al *[23]. Here, at each larval sampling station, the larval sampling area that would be shaded by the riparian vegetation when the sun is directly overhead was estimated by drawing on a tracing paper as above and then superimposed the drawings on the percentage shading diagrams from Nichols *et al*, and the proportion shading was estimated [23]. Measurements were taken monthly at all larval sampling stations between 11:00 and 13:00 h.

Salinity was measured *in situ *with a Hand Held Refractometer (Atago Co.Ltd, Japan). One drop of surface water from each larval sampling station was placed on the prism face of the refractometer and the reading on the scale was taken. This procedure was repeated 3 times and the values were averaged.

### Statistics

The association of the spatial factors (individual streams and the larval sampling stations), and the temporal factor (time of the collection - month) with anopheline larval presence was assessed with a generalized linear model (GLM) with a binomial distribution and a logit link function. The influence of six environmental factors on the presence and density of anopheline larvae was analyzed using generalized estimating equations (GEE) with large dammed streams, larval sampling stations and month as subject variables to account for repeated sampling. The six environmental factors were: filamentous algae, emergent aquatic plants, current rainfall, rainfall lagged by one month, salinity and sun exposure. The data was analyzed with two different distributions: 1) binary data (presence or absence) was fitted to a binomial distribution with a logit link function and 2) count data was fitted to a negative binomial distribution with a log link function because data was not normally distributed. For the density analysis, all larval collecting stations with zero counts of larvae were excluded. The level of significance for all tests was set at α = 0.05. All analyses were conducted with SPSS ver 17.

## Results

A total of 2,930 *An. farauti *larvae were collected of which 83% (n = 1,932) were early instars (I-II) and 17% (n = 499) late instars (III-IV). Of the 459 specimens reared successfully to adults, all were identified morphologically as *An. farauti *s.l. and verified by PCR-RFLP as *An. farauti*. There was no *An. punctulatus *or *An. koliensis *found (Table 1).

**Table 1:** Summary of the total number of *Anopheles farauti *larvae collected at Red Beach, Gilutae and Komuporo at Guadalcanal, Solomon Islands

Locality	Total number of larvae	3^rd ^and 4^th ^stage larvae	Number of eclosed adults	Mosquito identification	
				
				Morphology	PCR-RFLP
Red Beach	1046	221	193	*An. farauti *sl	*An.farauti ss*
Gilutae	750	175	167	*An. farauti *sl	*An.farauti ss*
Komuporo	1134	103	99	*An. farauti *sl	*An.farauti ss*
Total	2,930	499	459	*An. farauti *sl	*An.farauti ss*

### Larval presence and abundance in relation to spatial and temporal factors

**Figure 3:**
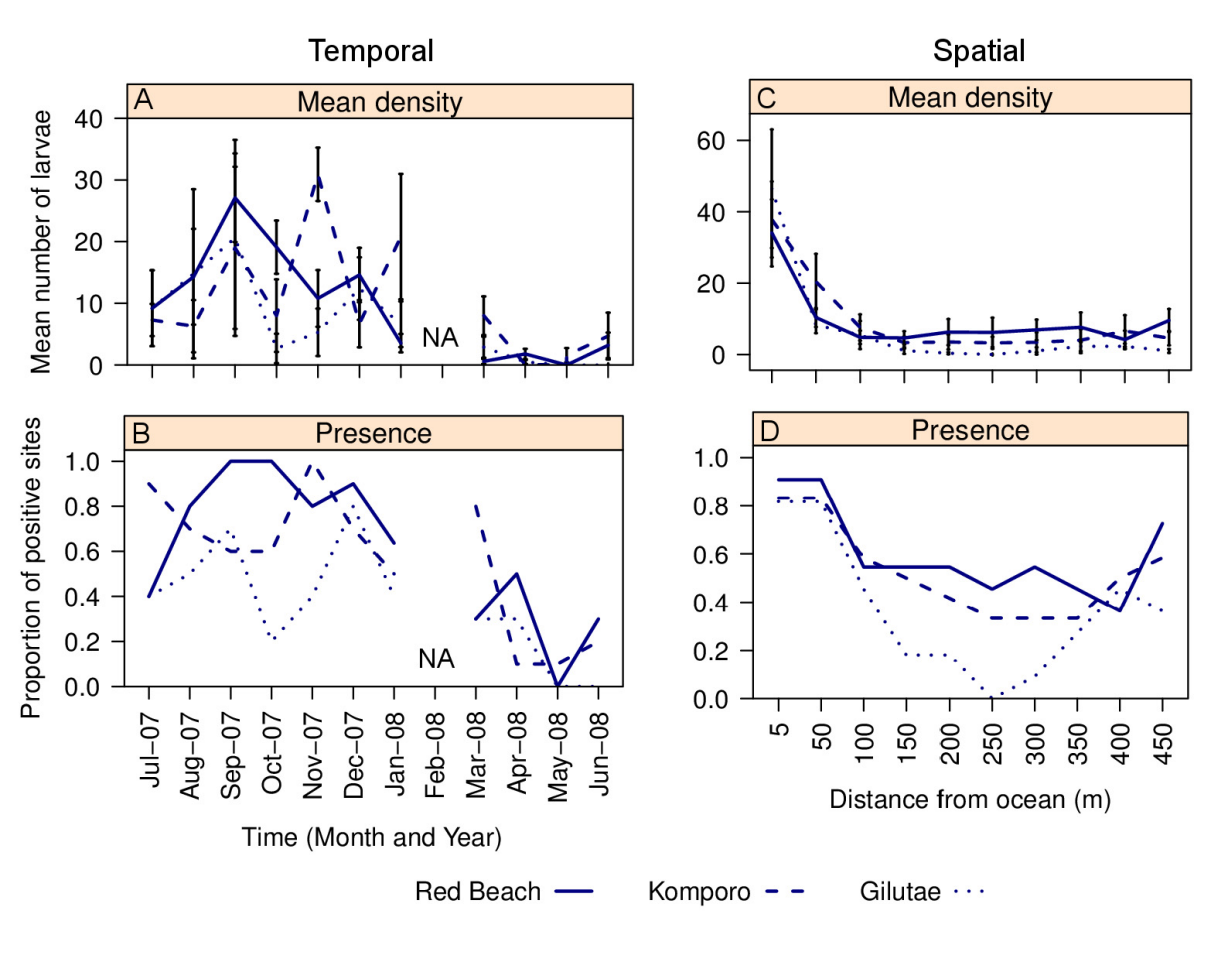
**A temporal and spatial comparison of mean larval mosquito density (A, C; mean ± SE) and the proportion of sites containing *An. farauti *larvae (B, D)**.

The spatial and temporal factors significantly influenced the presence or absence of *An. farauti *larvae: different times of the collections (χ^2 ^= 59.08, df = 10, p < 0.001), between the three streams (χ^2 ^= 22.32, df = 2, p < 0.001) and between the larval sampling stations (χ^2 ^= 45.18, df = 9, p < 0.001). Regarding time of collection, larvae occurred more often, and at significantly higher densities, during the dry season (July 2007 to December 2007) compared to the wet season (January to June 2008) (Figure 3A, B). In the wet season, when the sandbars were naturally removed and the streams were open to the ocean, the larvae were more susceptible to being flushed away during heavy storms whereas during the dry season with the large streams closed off there is little water flow. In the selected large streams (sites), the mean number of anopheline larvae from Gilutae, 6.82 ± 2.11 per 10 dips/station (total: 750) was lower than at Red Beach, 9.42 ± 1.77 per 10 dips/station (total 1046) and Komporo, 9.45 ± 1.77 per 10 dips/station (total: 1134). The larval sampling stations, that were located within 50 meters from the sea had significantly more larvae than the upstream larval sampling stations (Figure 3 C, D).

### Environmental factors in relation to spatial and temporal factors

**Figure 4:**
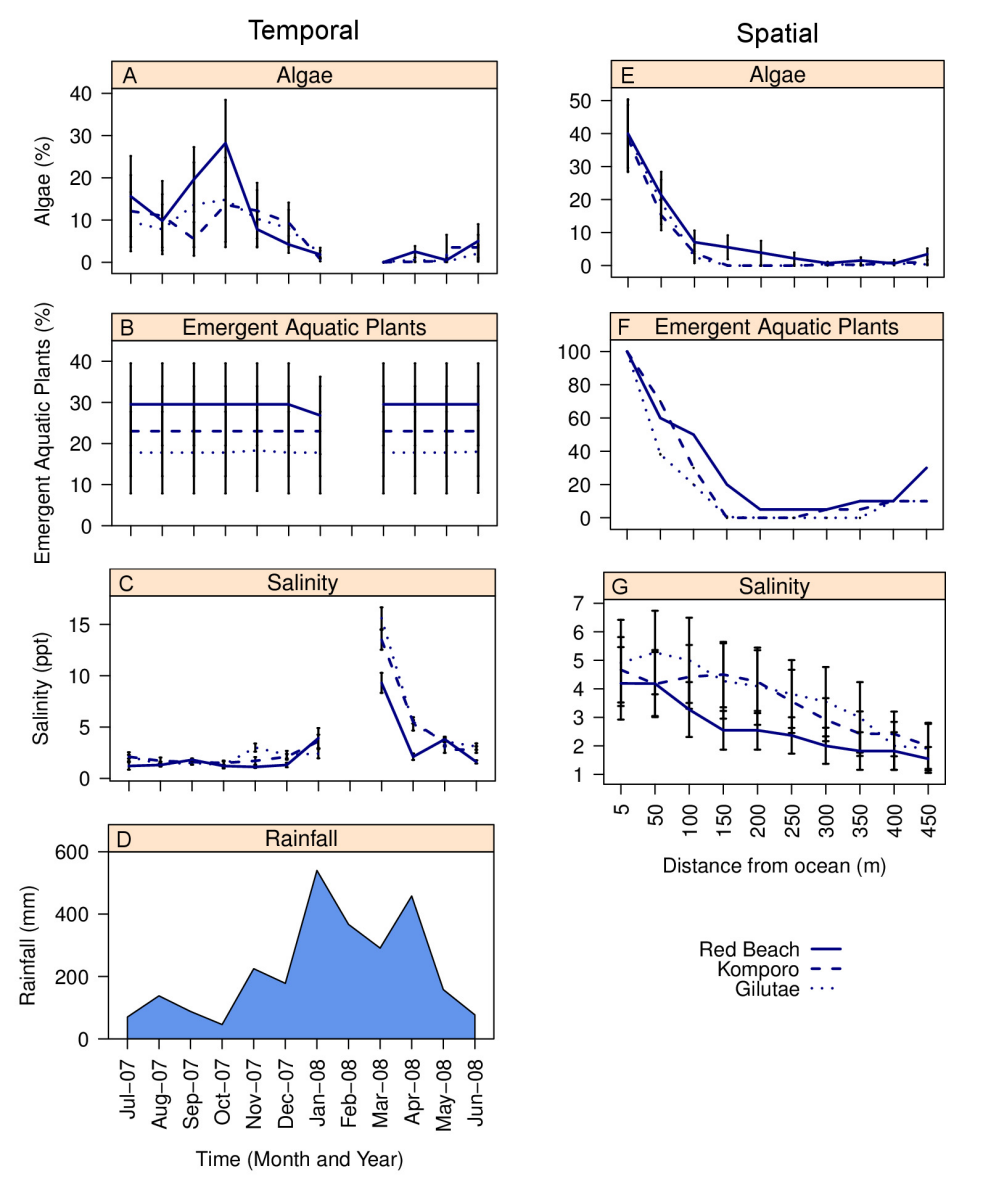
**A temporal and spatial comparison of the environmental factors recorded the study streams: filamentous algae (A, E; mean ± SE), emergent aquatic plants (B, F; mean ± SE), salinity (C, G; mean ± SE) and rainfall (D; monthly total)**.

Both spatial and temporal factors influenced the microclimate of the streams. Regarding temporal changes, the percentage algal cover was highest during the dry season compared to the wet season (Figure 4 A). Mean salinity ranged from 2‰ to 5‰ during the dry season, but was > 10‰ when the streams mouths were open during the wet season (Figure 4C). In contrast, there was no temporal difference in the percentage aquatic emergent plants (Figure 4D). Regarding spatial differences between the sampling stations, the percentage of algae and aquatic emergent plants and salinity was highest at stations less than 50 m from the sea (Figure 4 E, F, G).

### Larval presence and environmental factors

**Figure 5:**
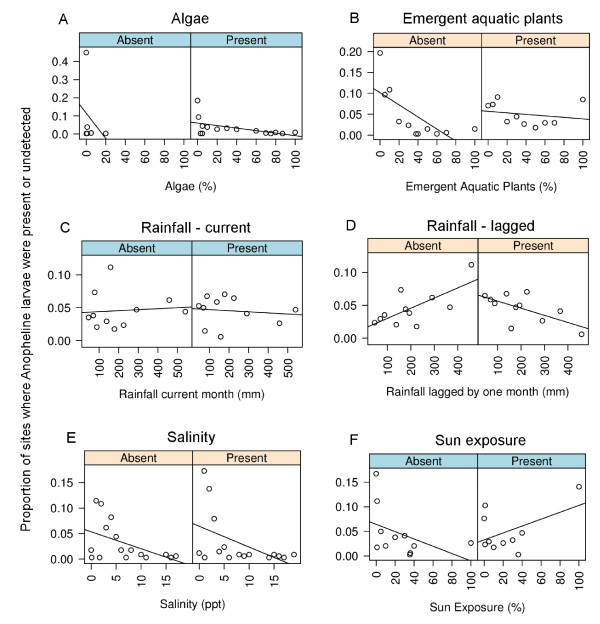
**Correlations between larval *An. farauti *presence and the 6 environmental factors in the study streams: filamentous algae, emergent aquatic plants, current rainfall, rainfall lagged by one month, salinity and sun exposure**. The factors with a pink top-panel were significantly associated with *An. farauti *presence (see Table 2).

The influence of the six environmental factors with larval presence or absence was assessed (Figure 5). Three factors significantly influenced larval presence (Table 2): emergent aquatic plants (p = 0.049), rainfall lagged by one month (p < 0.001) and salinity (p = 0.002). Emergent aquatic plants had a positive association with the presence of larvae, meaning that as the percentage of plants increased so did the chance of finding larvae. Rainfall, lagged by one month, had a negative association with larvae, when rainfall was high there was a lower chance of finding larvae the following month. With salinity, larvae were most commonly associated with brackish water being salinity readings ranged between 2‰ and 5‰ (Figure 5E). The factors that did not influence larval presence were: filamentous algae, sun exposure and current rainfall (Table 2, Figure 5).

**Table 2:** Association of environmental parameters with the presence and density of *An. farauti *larvae in brackish water streams in North Guadalcanal, Solomon Islands

Parameter	B	Std. Error	Chi-Square	*p *value
**1. Binary (presence/absence) model**				
Algae	-0.365	0.2284	2.550	0.110
Emergent Aquatic Plants	-0.019	0.0095	3.887	0.049*
Rain_current	-0.001	0.0007	0.773	0.379
Rain_lag	0.007	0.0012	35.116	0.000*
Salinity	-0.113	0.0374	9.195	0.002*
Sun Exposure	0.007	0.0077	0.889	0.346
**2. Negative binomial (density) model**				
Algae	0.023	0.0063	13.395	0.000*
Emergent Aquatic Plants	0.014	0.0040	11.783	0.001*
Rain_current	0.000	0.0006	0.291	0.589
Rain_lag	-0.008	0.0008	93.786	0.000*
Salinity	0.093	0.0376	6.072	0.014*
Sun Exposure	0.002	0.0042	0.295	0.587

Data were compared with GEEs with brackish water streams, larval sampling stations and month as subject variables to account for repeated sampling. The data was analyzed with two different distributions: 1) binary data (presence or absence) was fitted to a binomial distribution with a logit link function and 2) count data were fitted to a negative binomial distribution with a log link function because data was not normally distributed. For the density analysis, all larval collecting stations with zero counts of larvae were excluded.

### Larval density and environmental factors

**Figure 6:**
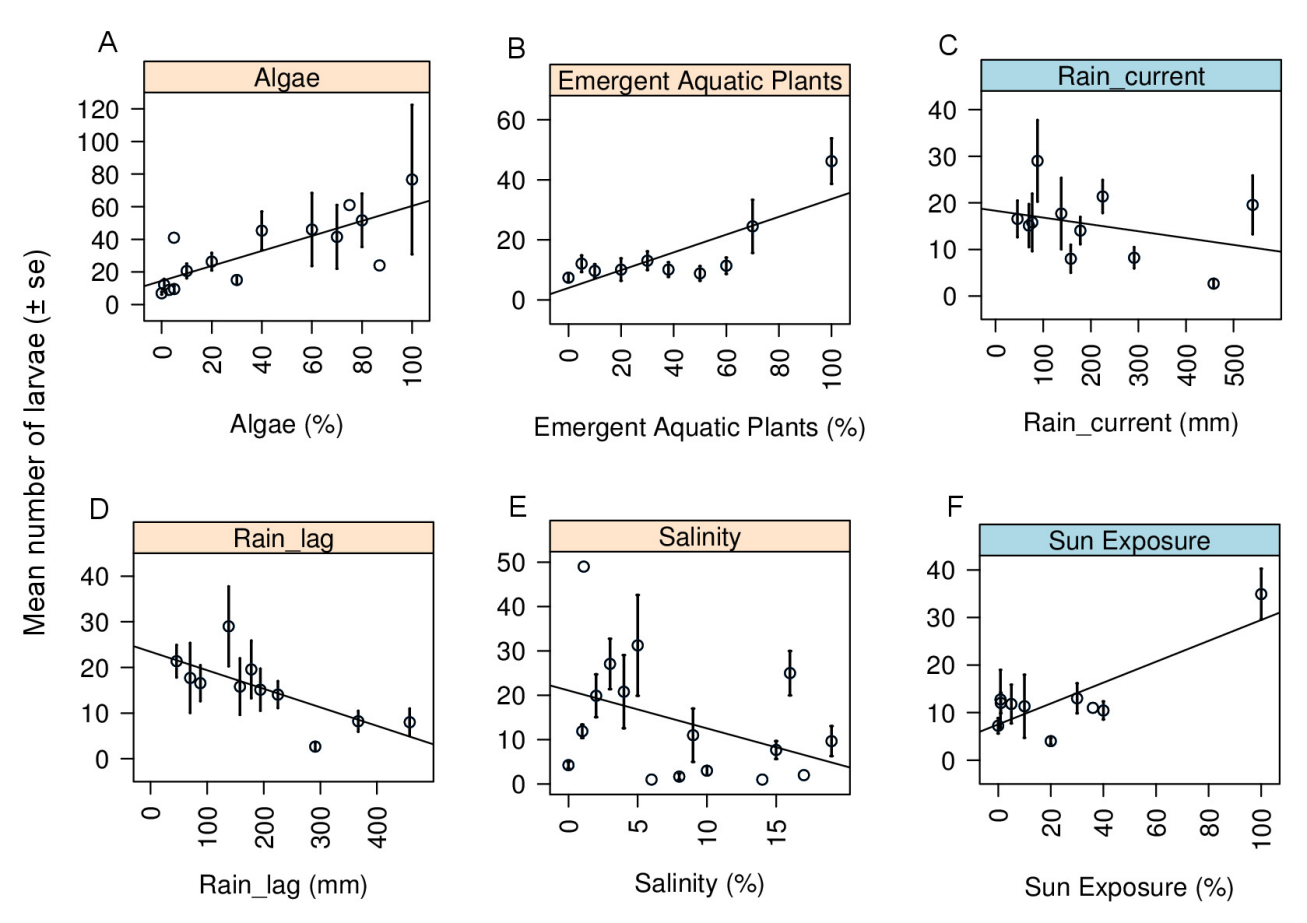
**Correlations between larval *An. farauti *density and the 6 environmental factors in dammed brackish water streams: filamentous algae, emergent aquatic plants, current rainfall, rainfall lagged by one month, salinity and sun exposure**. The factors with a pink top-panel were significantly associated with *An. farauti *density (see Table 2).

The three factors that influenced the presence of larvae also significantly influenced the density of larvae (Table 2) with emergent aquatic plants being positively associated (p = 0.001), rainfall lagged by one month being negatively associated (p < 0.001) and salinity also negatively associated (p = 0.014) (Figure 6). Additionally larval density was significantly influenced by filamentous algae (p < 0.001). Filamentous algae had a positive association with the density of larvae, meaning that as algal coverage increased so did the density of larvae (Figure 6). The factors that did not influence larval density were: current rainfall and sun exposure (Table 2, Figure 6).

## Discussion

This study demonstrated that the presence and density of *An. farauti *larvae varied significantly between large streams, larval sampling stations, and the time of sampling. Within individual streams, larval densities were highest at stations near the ocean where the water was generally brackish (2-5‰ salinity) and the coverage of filamentous algae and emergent aquatic plants was highest. This corresponds with previous observations made in the same region of Guadalcanal (Vutu, 500 m east of Gilutae) where larval densities were highest at the stream mouth and decreased inland [24]. Larval presence and density varied seasonally and was primarily driven by rainfall and the opening of the stream mouth. When rainfall was high, the sandbar at the mouth of the streams were washed away and in the following month the presence and density of larvae was lower. Similarly, severe rainfall has previously been associated with reduced adult densities of *An. farauti *in Papua New Guinea [25] and also larval densities of *Anopheles gambiae *in Kenya [26].

*An. farauti *larval presence and abundance was negatively associated with salinity, with the most occurrences and densities of larvae occurring in brackish water (2 - 5‰ salinity), conversely high salinities (10 - 28‰ salinity) were associated with lower presence and densities of larvae. In the wild, *An. farauti *has been found breeding in water ranging from fresh to seawater (35‰), however the conditions influencing the selection of these sites was unknown as was the successful emergence of adults [27]. Laboratory studies have indicated that oviposition of *An. farauti *will occur in water ranging from fresh to 35‰ salinity [8,28] however in choice studies there was a preference for fresh [28] or brackish water (< 50% seawater) [8]. Further laboratory studies have indicated that larval survival, from first instar, does not differ between fresh water and 50% seawater (96-92% survival) [9] and that survival from egg through to adults only occurs at < 65% seawater [8]. The work reported here is the first time that a detailed field study of larval salinity tolerance has been conducted longitudinally and while the salinity tolerance results generally concur with previous laboratory findings [9,10] there was a preference with the *An. farauti *population for lower salinities of 2-5‰ [5-14% seawater], although this may be confounded by the high percentage of algae and emergent aquatic plants also found at the stream mouth.

Filamentous algae and aquatic emergent plants were important predictors for *An. farauti *presence and abundance. Some *Anopheles *species are known to exhibit thigmotaxis, a tendency to maintain bodily contact with solid objects [1] and are rarely found in open water. In the streams studied here, the algae and emergent aquatic plants were able to satisfy this requirement for *An. farauti*. The presence of vegetation also provides a food source and some protection from predators and currents [8]. Observations that *An. farauti *is associated with vegetation have been reported in Solomon Islands and Vanuatu [5,8,29]. Positive association with vegetations have also been reported for *An. gambiae *in Kenya [30,31] and *Anopheles pseudopunctipennis *in Belize [32]. Filamentous algae have also been implicated in attracting gravid *Anopheles *females for oviposition [33].

Sun exposure was not an important predictor for *An. farauti *larval occurrence and abundance. This result contradicts previous findings that *An. farauti *prefer to breed in a more exposed environment compared to other anophelines of the *Anopheles lungae *complex [13]; however these observations were made prior to the use of molecular-based identification of *An. farauti *complex members and the observations may be confounded by the presence of *Anopheles hinesorum *or *Anopheles irenicus*, two other members of the *An. farauti *complex which occur in the Solomon Islands and which are morphologically very similar to *An. farauti*. In Vanuatu, where only *An. farauti *occurs, this species showed no preference for either shaded or sunlit sites, being commonly found in either [8]. The degree of exposure to sunlight is difficult to determine and interpret with regards to the occurrence of larvae. Breeding sites such as these dammed streams with surrounding vegetation and thick emergent vegetation will be shaded at some times of the day. As oviposition occurs at night, selection of either shaded or sunlit sites by gravid adults would not be possible and it would be the larvae themselves that select the degree of exposure to sunlight if such selection is important. It is more likely that a solid surface to rest against, avoidance of predators, and food requirements result in the larvae being in filamentous algae and aquatic emergent plants which always provide some shade.

This ecological data indicating where *An. farauti *larvae is most likely to be found and in what densities, is essential for designing cost-effective and evidence-based vector control programmes. Considering that these large streams are so highly productive, but relatively few in number, they present an opportunity where larval control could be targeted to the prime breeding habitats of *An. farauti*. The two most feasible options for targeting such highly productive large naturally dammed streams are: environmental manipulation or larviciding. Biological control using predacious fish would be ineffective as these streams are already stocked with local fish species which do not appear to have any impact on the larval populations most likely due to the protection offered by the vegetation.

With regards to environmental manipulation, managing the salinity and/or algae or plants in the system may be able to reduce the *An. farauti *larval population in such large dammed streams. It may be possible to manipulate the salinity and water flow of the streams by manually removing the sand bar. This would allow sea water into the site at high tides and also increase water flow removing vegetation from along the edges of the site and reducing suitable oviposition sites and larval harborage [15]. However, the mouth of the streams might be blocked again by sand migration in the dry season. For example, in Vanuatu *An. farauti *larvae and adults were eliminated from an entire village when one of these large dammed streams was naturally opened up to the sea, however when tides reestablished the sand dunes blocked the mouth and *An. farauti *quickly returned [8]. Alternatively, high density plastic pipelines could be installed underneath the sandbar to introduce the sea water into the dammed brackish water stream, but this technique is expensive and also requires constant maintenance. Other studies have manually manipulated the density of algae and plants in the environment to successfully reduce larval abundance; for example this has been seen in Mexico with the of immature stages of *An. pseudopunctipennis *[34,35]. However such methods would be financially expensive, logistically difficult and maintenance is on-going.

The best practical control method would be using a slow release larvicide, for example insect growth regulators. Recent evidence from both Africa and Asia has demonstrated empirically [36-40] and theoretically [41,42] that larviciding has potential to be an extremely successful vector control tool. For the current situation, granular formulations of a residual bio-larvicide could be placed along the edges of the brackish water streams (in some type of open weave bag). This study has established associations between environmental parameters and larval presence and abundance which would be useful in determining how extensive baiting would have to be to protect the people in the surrounding villages.

## Conclusion

The present study described for the first time in Solomon Islands, the relationship between larvae occurrence and density with environmental factors. Obviously there are subtle microclimates preferred during larval development including, a positive association with filamentous algae and aquatic emergent plants, while salinities over 10% seawater showed a negative association with larval occurrence and density, as did rainfall and the wet season. Overall the findings in this study support the notion that larval control is a feasible option for vector control that could complement the wide-scale use of LLINs and IRS in the region. Tools such as larval control and source reduction present an excellent opportunity to build complementary integrated vector control programmes that will be able to hit the vector at different stages of the life cycle, something that will be essential for the Solomon Islands where the primary vector *An. farauti *tends to be exophagic and exophilic. There is a need for further research into the efficacy of various larval control options for use in North Guadalcanal; however our results indicate that environmental manipulation or larviciding are both feasible options.

## Competing interests

The authors declare that they have no competing interests.

## Authors' contributions

HB, JH conceived the study. HB, TLR, RDC analyzed the data. HB, RDC, TLR, CCC wrote the manuscript. HB, JH, CB, CI, AA, CCC performed the field collections. HB, CCC performed the molecular analysis. JH, RDC, BKKC, AB, TLR reviewed the manuscript. All authors read and approved the final manuscript.
